# sRNAPipe: a Galaxy-based pipeline for bioinformatic in-depth exploration of small RNAseq data

**DOI:** 10.1186/s13100-018-0130-7

**Published:** 2018-07-31

**Authors:** Romain Pogorelcnik, Chantal Vaury, Pierre Pouchin, Silke Jensen, Emilie Brasset

**Affiliations:** GReD, Université Clermont Auvergne, CNRS, INSERM, BP 10448, 63001 Clermont-Ferrand, France

**Keywords:** Small RNA sequencing, sRNA-Seq, Galaxy, Bioinformatics analyses, Pipeline

## Abstract

**Background:**

The field of small RNA is one of the most investigated research areas since they were shown to regulate transposable elements and gene expression and play essential roles in fundamental biological processes. Small RNA deep sequencing (sRNA-seq) is now routinely used for large-scale analyses of small RNA. Such high-throughput sequencing typically produces several millions reads.

**Results:**

Here we present a computational pipeline (sRNAPipe: small RNA pipeline) based on the Galaxy framework that takes as input a fastq file of small RNA-seq reads and performs successive steps of mapping to categories of genomic sequences: transposable elements, gene transcripts, microRNAs, small nuclear RNAs, ribosomal RNAs and transfer RNAs. It also provides individual mapping and counting for chromosomes, transposable elements and gene transcripts, normalization, small RNA length analysis and plotting of the data along genomic coordinates to build publication-quality graphs and figures. sRNAPipe evaluates 10-nucleotide 5′-overlaps of reads on opposite strands to test ping-pong amplification for putative PIWI-interacting RNAs, providing counts of overlaps and corresponding z-scores.

**Conclusions:**

sRNAPipe is easy to use and does not require command-line or coding knowledge. This pipeline gives quick visual and quantitative results, which are usable for publications. sRNAPipe is freely available as a Galaxy tool and via GitHub.

**Electronic supplementary material:**

The online version of this article (10.1186/s13100-018-0130-7) contains supplementary material, which is available to authorized users.

## Background

One of the most significant biological discoveries of recent decades is the evidence that almost the whole genome is transcribed [1–3] and that most of the RNA molecules produced are less than 200 nucleotides (nt) long and correspond to multiple classes of small non-coding RNAs. The small non-coding RNAs include both housekeeping RNAs, such as transfer RNAs (tRNAs) and ribosomal RNAs (rRNAs), and regulatory RNAs, such as microRNAs (miRNAs), small nuclear RNAs (snRNAs), PIWI-interacting RNAs (piRNAs) and small interfering RNAs (siRNAs).

Different pathways of RNA interference, which are conserved in eukaryotes, generate these small regulatory RNAs. The biogenesis of siRNAs and miRNAs is dependent on the double-stranded RNA-specific ribonuclease Dicer, whereas piRNA biogenesis is not. The various types of small regulatory RNAs, with lengths ranging from 20 to 30 nt, guide Argonaute family proteins to RNA targets and regulate expression of diverse sequences. Non-coding RNAs are the subject of extensive studies and have been reported to play key roles in most cellular processes. Indeed, by a mechanism of transcriptional or post-transcriptional repression, these regulatory non-coding RNAs are key molecules in gene expression regulation, antiviral defence and defence against massive mobilization of transposable elements (TEs), highly mutagenic mobile DNA sequences. Whereas miRNAs are more dedicated to regulate gene expression, siRNAs and piRNAs function to repress TEs and maintain genome stability of the somatic and the germ line cells respectively [4–6].

The abundance and the repetitive nature of these molecules add another layer of complexity to the small RNA analysis. Consequently, there is an increasing need for tools orchestrating the analysis to ensure repeatability and standardization of the processing and to reduce repetitive effort performed by bioinformaticians. Galaxy, which is a popular application for bioinformatics analyses, is an excellent web-based interface to wrap up a pipeline performing small RNA-seq analysis. With that in mind, we have developed sRNAPipe, a pipeline to perform successive steps of small RNA mapping, counting, normalization, drawing publication-quality figures by plotting reads along genomic coordinates, and analysis of eventual signatures for ping-pong amplification in the case of piRNAs.

## Implementation

The initial input of the sRNAPipe is a collection of single-end sequencing data in a fastq phred+ 33 format, following adapter removal, and a list of input multi-fasta references (genome, transcripts, TEs, rRNAs, tRNAs, snRNAs, miRNAs). For species with a poor genome, the user may use any existing or combined input file as a “genome”. If rRNAs, tRNAs and snRNAs have not been annotated, the pipeline may be run without these input references.

The user can choose the size range of small RNAs that shall be explored and the size range of reads that the user considers as siRNAs or piRNAs, as well as maximal numbers of mismatches for alignment to the genome and to TEs.

**Fig. 1:**
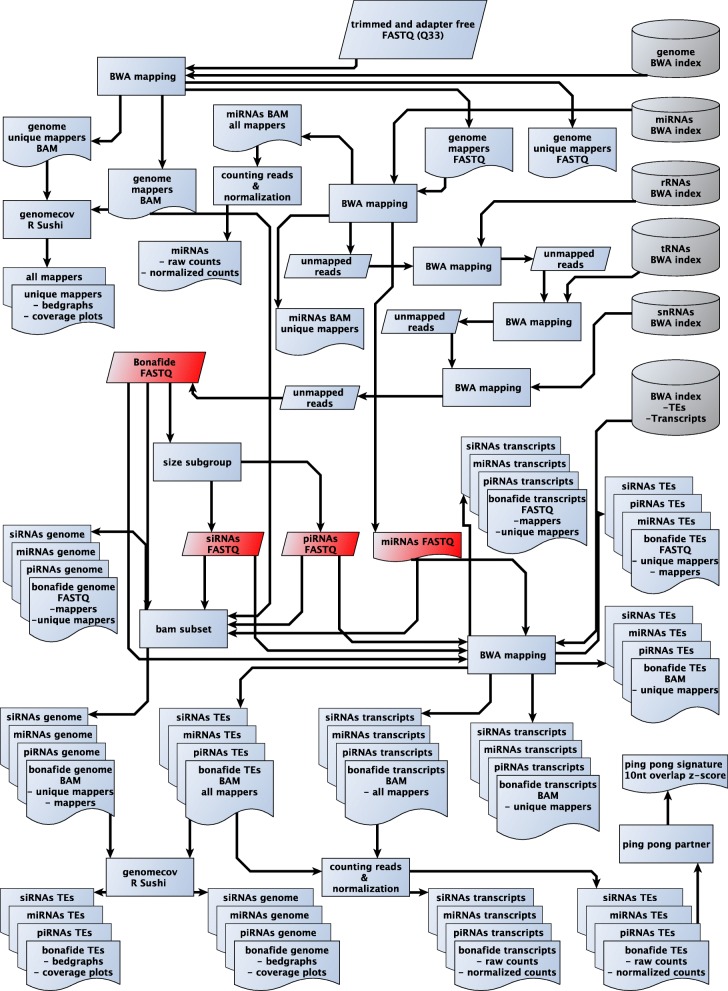
sRNAPipe workflow for an in-depth analysis of sRNA-seq data

First, sRNAPipe creates the BWA [7] index for all the input references (Fig. 1). After this step, all libraries of the collections are processed in parallel. Reads of the chosen length (default: 18–29 nt) are selected and aligned against the reference genome using BWA allowing up to the selected maximal number of mismatches. The result of this first step is a SAM (Sequence Alignment/Map) file output by BWA sorted by genomic position and filtered out for unmapped reads using SAMtools [8]. The corresponding bedgraphs are created using BEDtools genomecov [9]. The R/Bioconductor package Sushi [10] is used to plot the coverage along the chromosomes or transposable elements.

For the following steps, only the genome-mapping reads with the selected maximal number of mismatches are conserved and for accurate mapping of small RNA reads, ribosomal RNA, miRNA, tRNAs, snRNAs filtering was performed before mapping the resulting bonafide reads on transcripts and TEs. After each mapping, only unmapped reads are kept for the next alignment. Reads mapping to each category of reference are counted to create a pie chart displaying the distribution of genome-mapping small RNAs (percentage relative to total mapped reads).

At the end of this step, four subgroups are selected for further analysis:

- Bonafide reads: correspond to the genome-mappers excluding reads that map miRNAs, rRNAs, tRNAs or snRNAs.
- miRNAs: genome-mappers matching miRNA reference.
- siRNAs: Bonafide reads of user-selected length (default: 21 nt)
- piRNAs: Bonafide reads of user-selected length (default: 23 to 29 nt)

For each subgroup, sorted BAM files for all the genome-mappers and for the genome-unique mappers, bedgraphs for the plus and minus strand mappers as well as the corresponding graphs of the genome alignment are created.

In the miRNA subgroup, a list of the miRNAs referenced in the corresponding multi-fasta input file and their number of reads are reported, as well as the corresponding normalized values: in RPKM (Reads Per Kilobase per Million of mappers), reads per million of piRNAs, miRNAs or bonafide reads.

Each of the four subgroups is in parallel mapped against transcripts and TE references allowing up to the selected maximal mismatch number. For each subgroup the sorted BAM files for all mappers and for genome-unique mappers, bedgraphs for the plus and minus strand mappers and the corresponding graphs are created. In addition, the counts of mapping reads per feature as well as the corresponding normalized values in RPKM, reads per million of piRNAs, miRNAs or bonafide reads are given in a table. For TEs, three tables are created: 1-Without mismatches, 2-with mismatches between zero and the user-defined maximal mismatch number, 3-with mismatches between 1 and the user-defined maximal mismatch number. In each table, for each individual TE or transcript, the percentage of reads with U and A residues at nucleotide positions 1 and 10 respectively (“1U”, “10A”), in both sense and orientation, is calculated. Indeed, a positive bias for these features indicates that a ‘ping-pong’ mechanism may be responsible for the generation and amplification of the corresponding piRNAs [5].

Finally, for small RNAs mapping to TEs (with mismatches between zero and the user-defined maximal mismatch number) in the piRNA subgroup, sRNAPipe analyses the ping-pong signature, which corresponds to a high frequency of 10-nt 5′-overlaps between reads mapped on opposite strands. In order to identify this signature, the number of 5′-overlaps from size 1 to the minimum piRNA read size chosen is computed and it is tested if the 10-nt 5′-overlap number fits into a normal distribution within all overlaps by calculating the z-score corresponding to the 10-nt 5′-overlaps. sRNAPipe also builds the corresponding histograms and furnishes the lists of piRNAs with ping-pong partner, i.e. with 10-nt 5′-overlap, or without such ping-pong partner.

Prerequisites and instructions to install and use sRNAPipe can be found in Additional files 1 and 2.

## Results and discussion

The results can be retrieved in the main html output page. On the first page, the number of all genome-mappers and the number of genome-unique mappers (reads that map only once to the genome) are reported and used later for normalization.

**Fig. 2:**
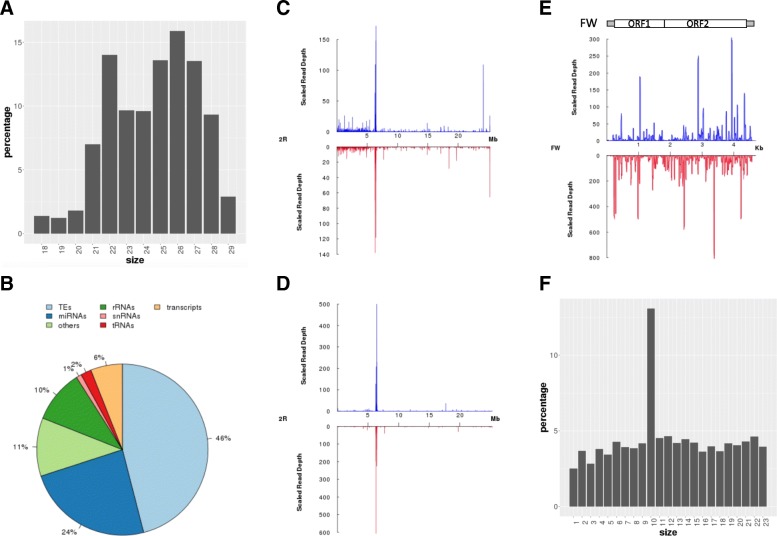
Examples of results obtained from sRNAPipe. **a** Length distribution of the reads. **b** Mapping to categories of genomic sequences. The pie chart shows percentages of reads of the chosen size range (here 18–32 nt) mapping to microRNAs (miRNAs), gene transcripts (“transcripts”), small nuclear RNAs (snRNAs), ribosomal RNAs (rRNAs), transfer RNAs (tRNAs), transposable elements (TEs) and other, non-annotated sequences (“others”). **c** Mapping of bonafide piRNAs, 23–29 nt long, along genomic coordinates of chromosome 2R. Reads mapped to the Plus strand are in blue, those mapped to the minus strand in red. **d** Mapping of genome-unique bonafide piRNAs, 23–29 nt long, along genomic coordinates of chromosome 2R. Colors as in C. **e** Density profile of putative piRNA coverage (23–29 nt) along the FW LINE-like retrotransposon, mapped allowing 0 to 3 mismatches. Colors as in C. **f** Histogram of the percentage of 5′-overlaps of sense and antisense read of the FW-mapping piRNAs (23–29 nt)

The length distribution of mapped reads is also provided, allowing to check whether it fits the expected length distribution (with the typical peaks according to the species). We evaluated our pipeline extensively and used it to analyse hundreds of small RNA-seq libraries with multiple configurations including different species, different genome releases and different RNA extraction protocols [11–14]. To present examples, the pipeline was run with the SRA dataset SRR4428936 [12]. Figure 2a depicts a bimodal size distribution, with a peak at 22 nt that represents the miRNA population and an enrichment of small RNAs with sizes between 23 and 29 nt which represents the piRNA population.

A pie chart represents the distribution of the genome-mappers in each category (Fig. 2b). This representation allows a quick visualization of the composition of the small RNA population. In the example, 46% of the genome-mappers correspond to TEs and 24% to miRNAs, 6% of the reads correspond to gene transcripts.

For each chromosome, scaffold or contig, two figures are created. They represent either all mappers or genome-unique mappers, normalized in RPKM (Reads per kilobase per million mappers), mapping on the plus strand or on the minus strand all along each chromosome. The chromosome arm 2R of *Drosophila melanogaster* is represented in Fig. 2c (all mappers) and d (genome-unique mappers). To address the exact genomic origin of these small RNAs, which are of repetitive nature, it is possible to restrict the analysis to only the small RNAs that map the genome at a unique position. This method, used by Brennecke et al. [5], allows identifying discrete loci where piRNAs are enriched, which were called piRNA clusters. One of the most predominant piRNA cluster (42AB) is visualized by the peak at 500–600 RPKM on the graph presented Fig. 2d.

The information for each of the four subgroups, Bonafide reads, siRNAs, piRNAs and miRNAs, is accessible via the index page clicking on “View details”. For each subgroup, three distinct analyses are accessible in different folders: for genome-mapping reads, TE-mapping reads and transcript-mapping reads. These folders contain tables with read counts as well as the percentage of reads with a 1U or 10A for the corresponding features (genome, TEs or transcripts), sorted BAM files for all mappers and for genome-unique mappers, bedgraphs for the plus and minus strand mappers. All these results can be downloaded. For small RNAs that map to TEs, the small RNAs are plotted all along the TE sequence in sense and in antisense orientation. piRNAs mapping the transposable element FW are represented in Fig. 2e as an example. It shows that both sense and antisense piRNAs of FW are produced in drosophila ovaries.

For piRNAs mapping TEs, the ping-pong signature is analysed for each TE and a sum of all overlaps, the sum of 10-nt-overlaps, the mean, the standard deviation, the z-score and the *p*-value for each TE are calculated and summarized in a table (Table 1). By clicking on a particular TE in the table, a histogram of the percentage of 5′-overlaps of reads in opposite orientation is accessed. Reads with or without ping-pong partners, in sense and in antisense orientation, can be downloaded for further analysis. As shown in Fig. 2f, the FW-mapping piRNAs (23–29 nt) are produced thanks to the ping-pong mechanism since an enrichment of 10-nt-overlaps is detected.

**Table 1:** Head of the table with the results of ping-pong signature analyses as an example

ID	Overlap sum	Ten overlap sum	Mean	Standard deviation	z-score	*p*-value
ACCORD2_I	762	39	33.1304347826087	10.5965718321339	0.553911709406997	0.289819635540828
ACCORD2_LTR	1267	166	55.0869565217391	39.3887128430581	2.81585854100354	0.00243235406595044
ACCORD_I	6312	579	274.434782608696	80.8309164307798	3.76792978280929	8.23034928753019e-05
ACCORD_LTR	973	207	42.304347826087	41.7200137381785	3.94764136961916	3.94624570775326e-05
BAGGINS1	153161	15635	6659.17391304348	2082.26495937923	4.31060708510045	8.14034819840437e-06
BARI1	13	0	0.565217391304348	1.01407859040788	−0.557370401713154	0.711362808127372
BARI_DM	10053	1018	437.086956521739	189.232066646377	3.06984462926058	0.00107085077343727
BATUMI_I	383752	42525	16684.8695652174	5914.46249756989	4.36897358727014	6.24159308748595e-06
BATUMI_LTR	4710	627	204.782608695652	118.651098502304	3.55847856980565	0.000186504609273053

For each TE, the table contains the sum of all overlaps, the sum of 10-nt-overlaps, the mean of all overlaps, the standard deviation, the z-score and corresponding *p*-value for 10-nt-overlaps

## Conclusions

sRNAPipe is a new Galaxy bioinformatics tool allowing a fast and user-friendly analysis of small RNA-seq data. sRNAPipe presents several advantages when compared to other tools such as Mississippi (Galaxy Project) or piPipes [15] which are respectively inside or outside the Galaxy environment. Indeed, sRNAPipe consists of a series of tools all wrapped together to get results in one simple, rapid and reproducible run, and without advanced computational skills. The user has the possibility of processing several sRNA libraries in parallel with the same parameters, thus easily allowing a comparison of the libraries and identification of eventual differences.

This pipeline allows to get very quickly visual and precise quantitative results since 15 millions of reads are analysed in approximately 2 h. This tool should be of interest to a broad community of researchers including not only scientists working on transposable elements and their control by the host but also the ones who work on the regulation of gene expression by non-coding RNAs or on any genomic sequences that produce small RNAs.

## Availability and requirements

Project name: sRNAPipe

Project home page: sRNAPipe is freely available via GitHub: https://github.com/brassetjensen/sRNAPipe, and in the Galaxy Toolshed: https://toolshed.g2.bx.psu.edu/repository?repository_id=13a327665795142c

Operating system(s): Unix

Programming language: Perl

Other requirements: Galaxy server

License: GNU GPL

Any restrictions to use by non-academics: none

## Additional files

Additional file 1: ReadMe. This file gives instructions concerning the prerequisites and the installation of sRNAPipe. (TXT 3 kb)

Additional file 2: sRNAPipe User Manual. User manual with extensive details and a step by step approach to easily use sRNAPipe. (DOCX 2730 kb)

## Abbreviations

miRNA: microRNA
nt: nucleotides
piRNA: PIWI-interacting RNA
RNA-seq: RNA sequencing
RPKM: Reads per kilobase per million mappers
rRNA: ribosomal RNA
siRNA: small interfering RNA
snRNA: small nuclear RNA
sRNA-seq: Small RNA deep sequencing
TE: Transposable element
tRNA: transfer RNA
