# Supplementary material for: sRNAPipe: a Galaxy-based pipeline for bioinformatic in-depth exploration of small RNAseq data
Source: Mob DNA. 2018 Jul 31;9:25. doi: 10.1186/s13100-018-0130-7 (PMC6069783; doi:10.1186/s13100-018-0130-7)
Supplement: Supplementary file 2 — sRNAPipe User Manual. User manual with extensive details and a step by step approach to easily use sRNAPipe. (DOCX 2730 kb) [file 13100_2018_130_MOESM2_ESM.docx]

Mobile DNA manuscript – **User Manual** –

"*sRNAPipe*: a GALAXY-based pipeline for bioinformatic in-depth exploration of small RNAseq data"

**Prerequisites to use *sRNAPipe*:**

The initial input of the *sRNAPipe* is a collection of single-end sequencing data in a fastq phred+33 format (Galaxy fastqsanger format), following adapter removal, and a list of input multi-fasta references (genome, transcripts, TEs, rRNAs, tRNAs, snRNAs, miRNAs, fasta format). If your uploaded file has not been recognized with the required format, the format can be changed using the Pencil icon to access Datatypes.

Sequencing data from Dennis C. et al. 2016, SRR4428936.fastq can be used to test the pipeline as in "sRNAPipe: a GALAXY-based pipeline for bioinformatic in-depth exploration of small RNAseq data", Pogorelcnik R, Vaury C, Pouchin P, Jensen S and Brasset E, Mobile DNA, 2018. Small artificial datasets (available via GitHub: https://github.com/brassetjensen/sRNAPipe) can be used to test the pipeline very quickly, within a few minutes (see below).

**To run *sRNAPipe*,**

1. Open *sRNAPipe* (in-depth study of small RNA) software in the Tools panel on the left.

**
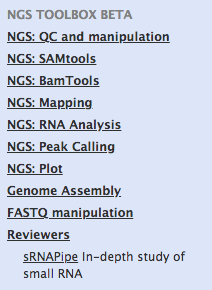
**

1. To run *sRNAPipe* with the sequencing data from Dennis C. et al. 2016, SRR4428936.fastq, enter all the parameters as described here:


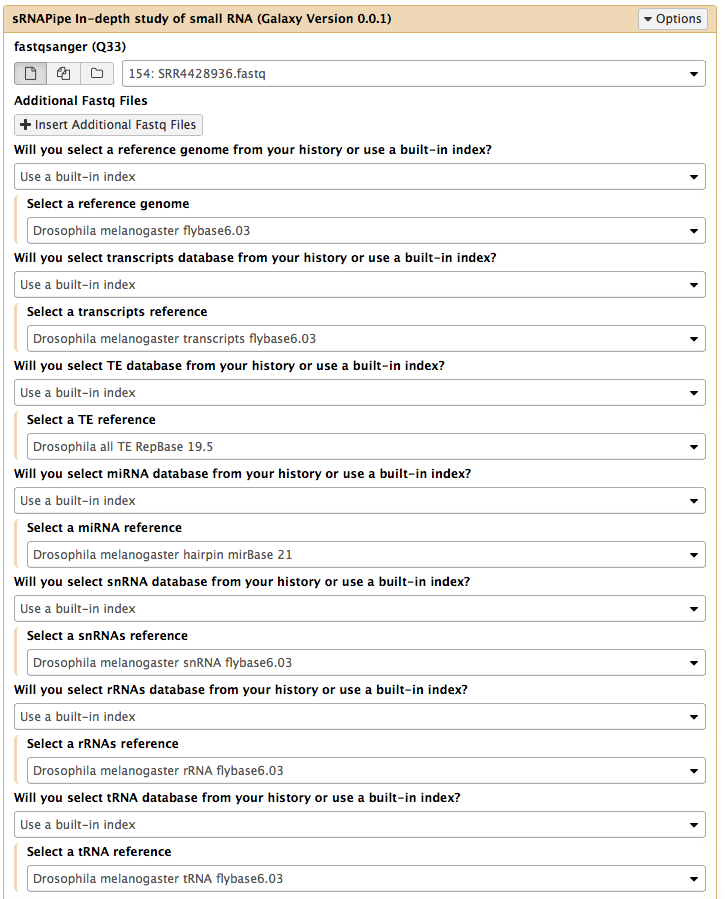


At least the sequencing data, genome, transcripts, TEs and miRNA files are required to run sRNAPipe. Files for snRNAs, rRNAs and tRNAs may be skipped by choosing "None" in answer to "Will you select snRNA/rRNAs/tRNA database from your history or use a built-in index?". If the user has no access to a microRNA dataset (e.g. non-model species), it is possible to use an empty "miRNA" file, containing only a fasta title and a sequence composed of a series of N's.

To run *sRNAPipe* with small test-data files (available via GitHub: https://github.com/brassetjensen/sRNAPipe) for a quick test, enter all the parameters as described here:


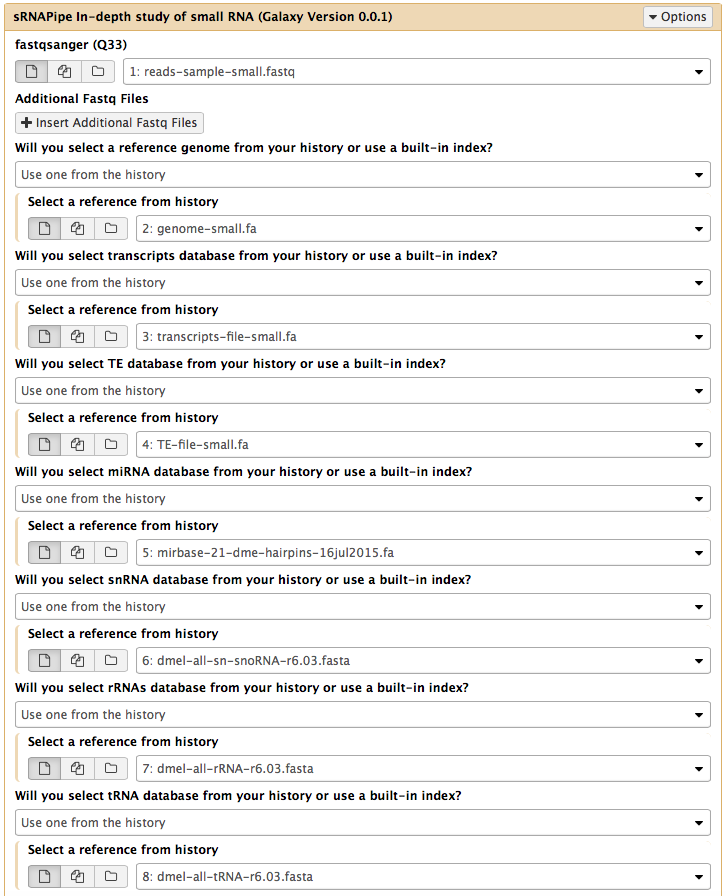


1.
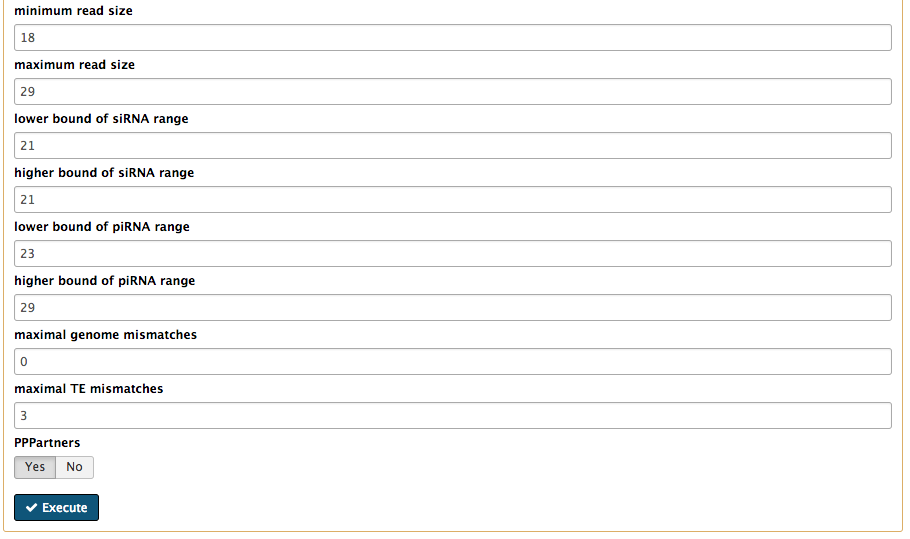
Select the minimum and maximum size of the reads to analyze, the size of the siRNA and piRNA
2.
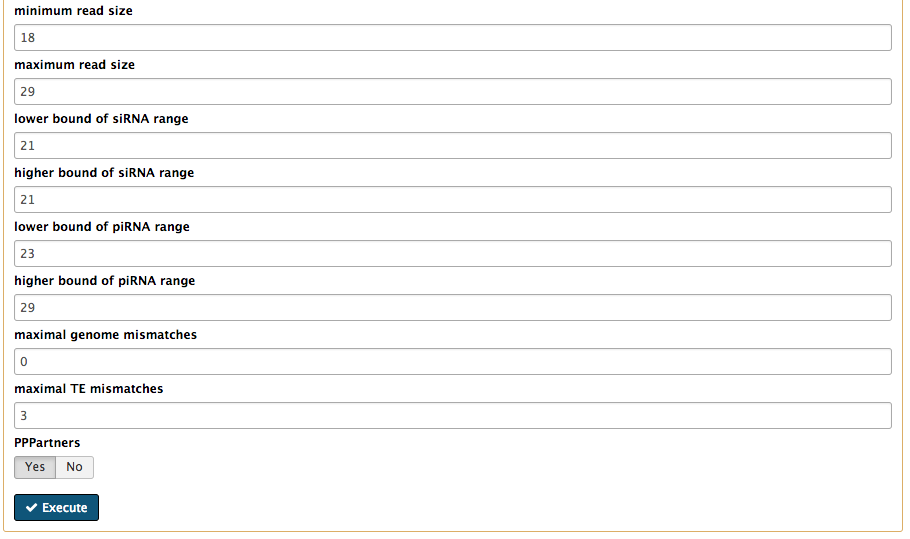
Choose the maximum number of mismatches allowed for the mapping on the genome and on the TEs, and whether you wish to study eventual ping-pong partners ("PPPartners"). Click on Execute.
3. Running job items appear in yellow, and when achieved in green.
4. A first results item “sRNAPipe_fastq_output” is created containing the 26 fastq files generated when the job is completed. Each of these files can be visualized by clicking on the name “sRNAPipe_fastq_output”, and downloaded for further analysis by clicking on its name and then on the Save icon. To come back to the History, click on "Back to <History name>"


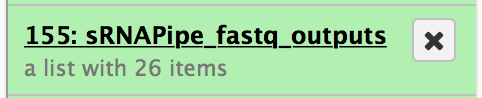

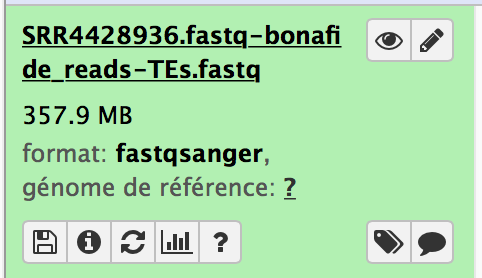

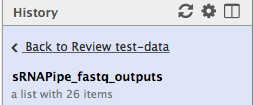


The “sRNAPipe_ fastq _output” files can be unhidden to be used for further analysis on the Galaxy platform by clicking on "hidden" in the History panel and then on "Rendre visible".


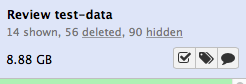

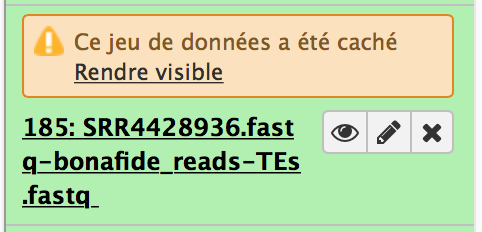


1. A second results item “sRNAPipe_<name input fastq file>” is created. The results can be viewed by clicking on the Eye icon.


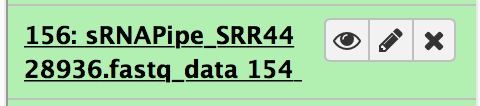


If you ran *sRNAPipe* with several fastq files, click on "Page" icon to view the respective results.


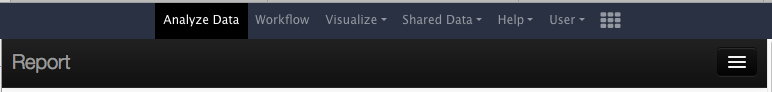


Note: If the user wants to analyze transcripts with the same details as TEs (coverage plots, ping-pong), the input TE database can be replaced with the transcripts database or a fasta file containing a subset of transcripts. In this case, instead of the "transcripts" and "miRNA" databases, the user should use "empty" fasta files, containing only a fasta title and a short sequence composed of a series of N's.

**Results as obtained with the sequencing data from Dennis C. et al. 2016, SRR4428936.fastq:**

1. On the first page:
   - The size distribution of genome-mapped reads and the number of all genome-mappers and of genome-unique mappers (reads that map only once to the genome) can be visualized.


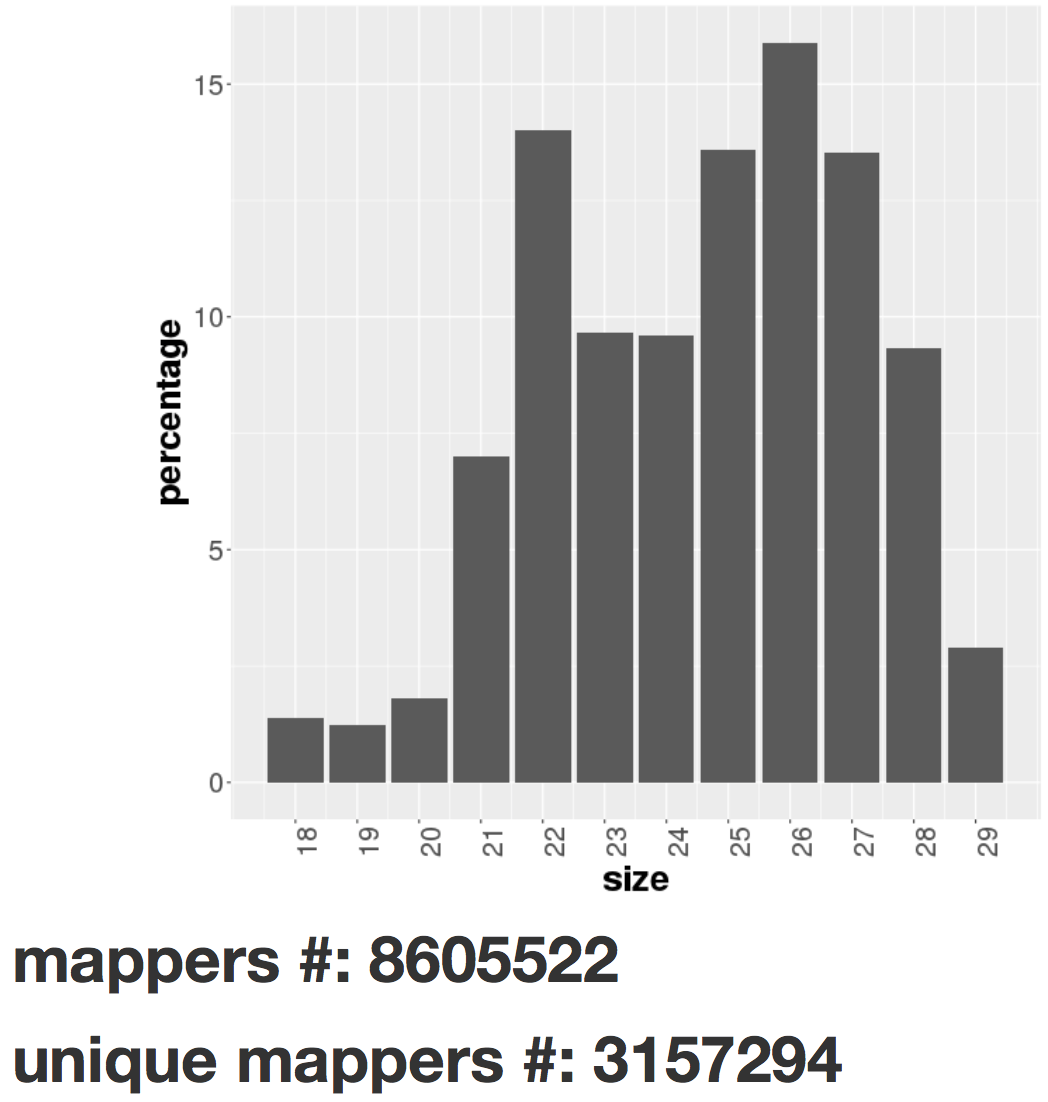


- - Figures show mapping of genome-unique reads ("Uniquely mapped reads") and of all reads randomly assigned ("Reads randomly assigned") to the chromosomes. Small RNAs are mapped on plus and on minus strands, in blue and in red respectively.


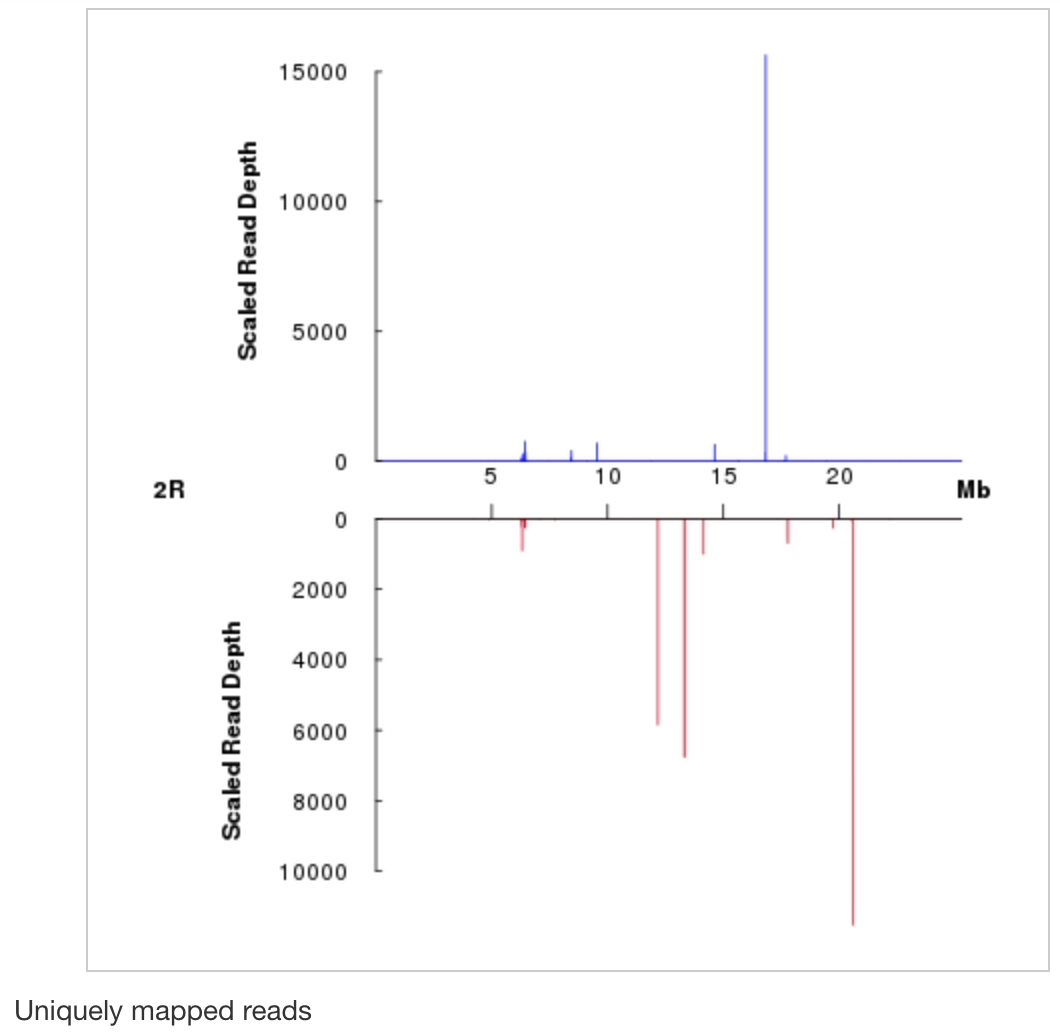

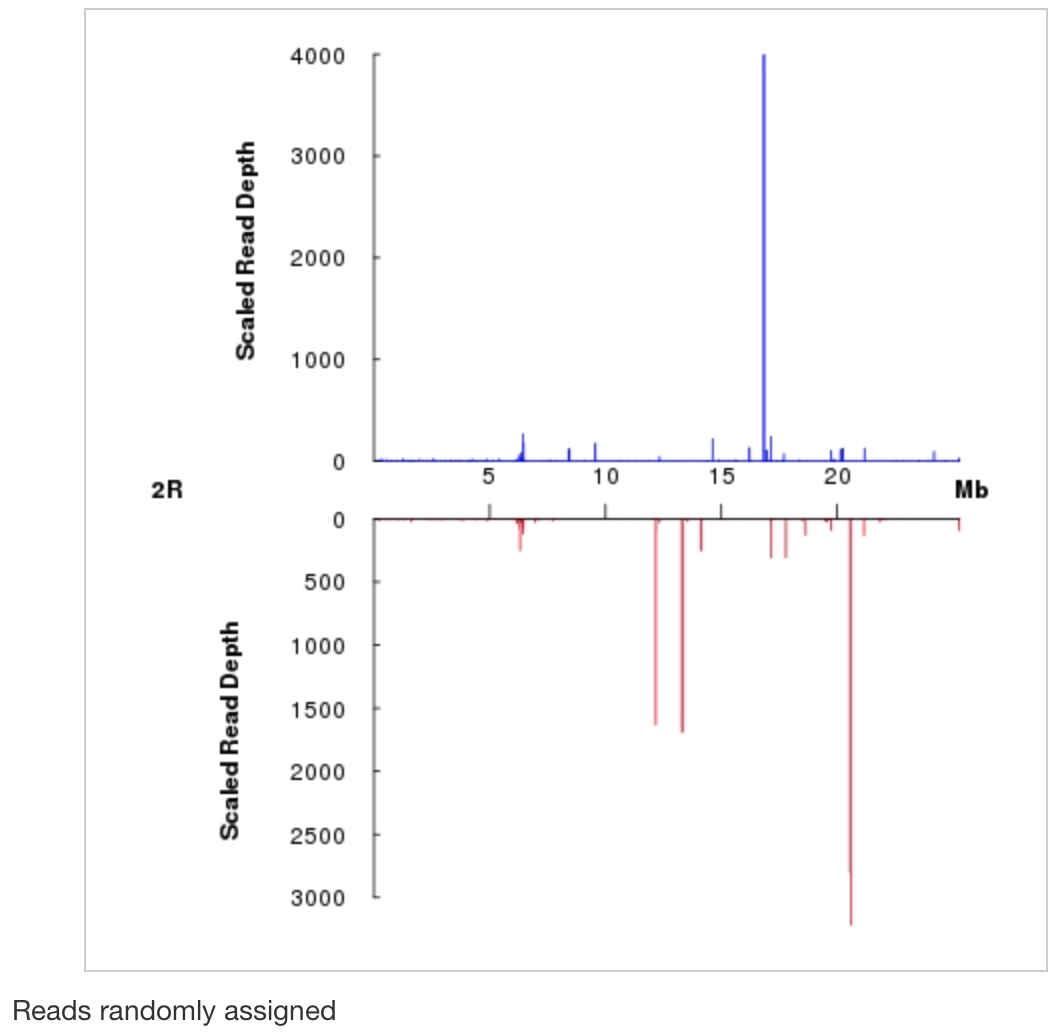


- -
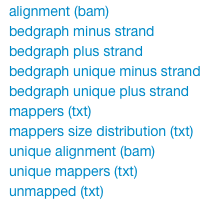
The corresponding bam and bedgraph files are accessible *via* the corresponding links on the upper left, as well as the counts of the mappers size distribution and lists of the mapped and unmapped reads.

1. The second page is accessible by clicking on "View details" on the first page of results. Then the information for 4 subgroups, Bonafide reads, siRNAs, piRNAs and miRNAs, is accessible. For each subgroup, three distinct analyses are accessible in different folders: for genome-mapping reads, TE-mapping reads and transcript-mapping reads.


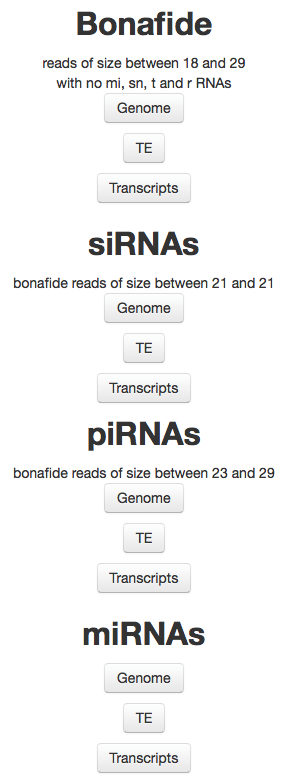


1. By clicking on Genome, TE or Transcripts folders, figures for genome-unique mappers and for all mappers and tables with read counts for the corresponding features (genome, TEs or transcripts) can be visualized, the corresponding sorted BAM files and bedgraphs for the plus and minus strand mappers can be downloaded. To quickly search for a desired TE or contig, a search function is available.

Example: Click on Bonafide and then Genome folder:


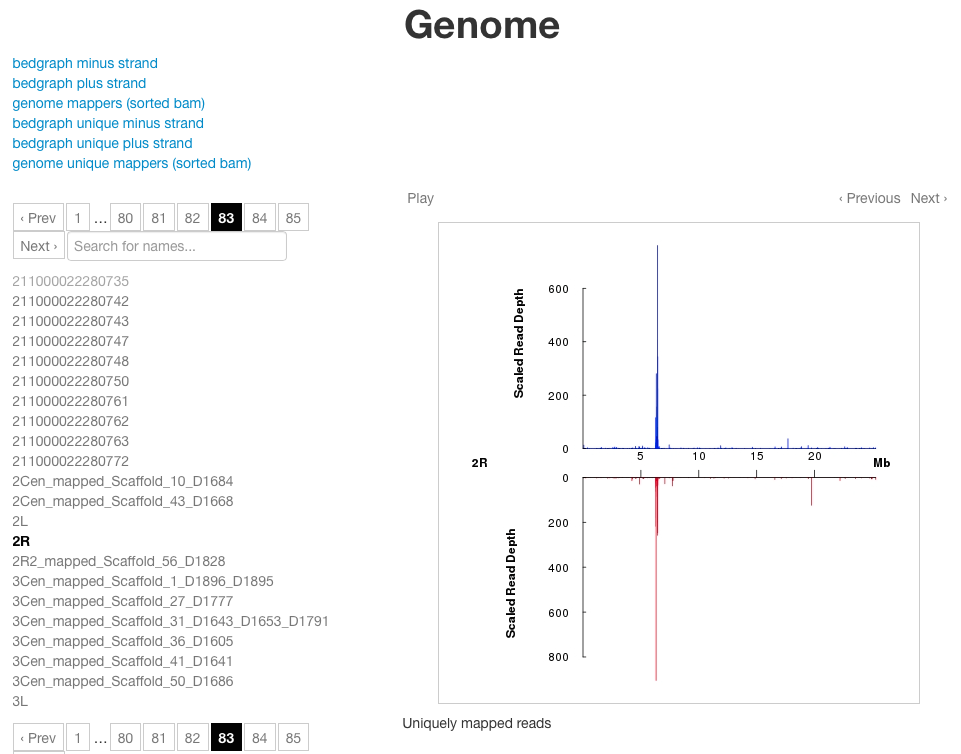

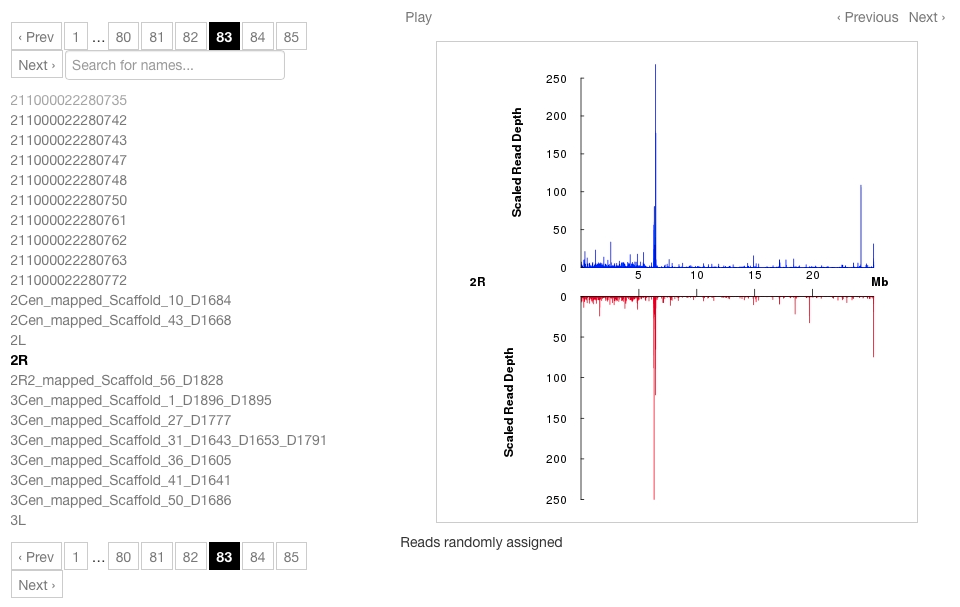


1. The results for Ping-pong partners are found only in the "piRNAs" subgroup "TE" folder, by clicking on "Ping Pong Partners".


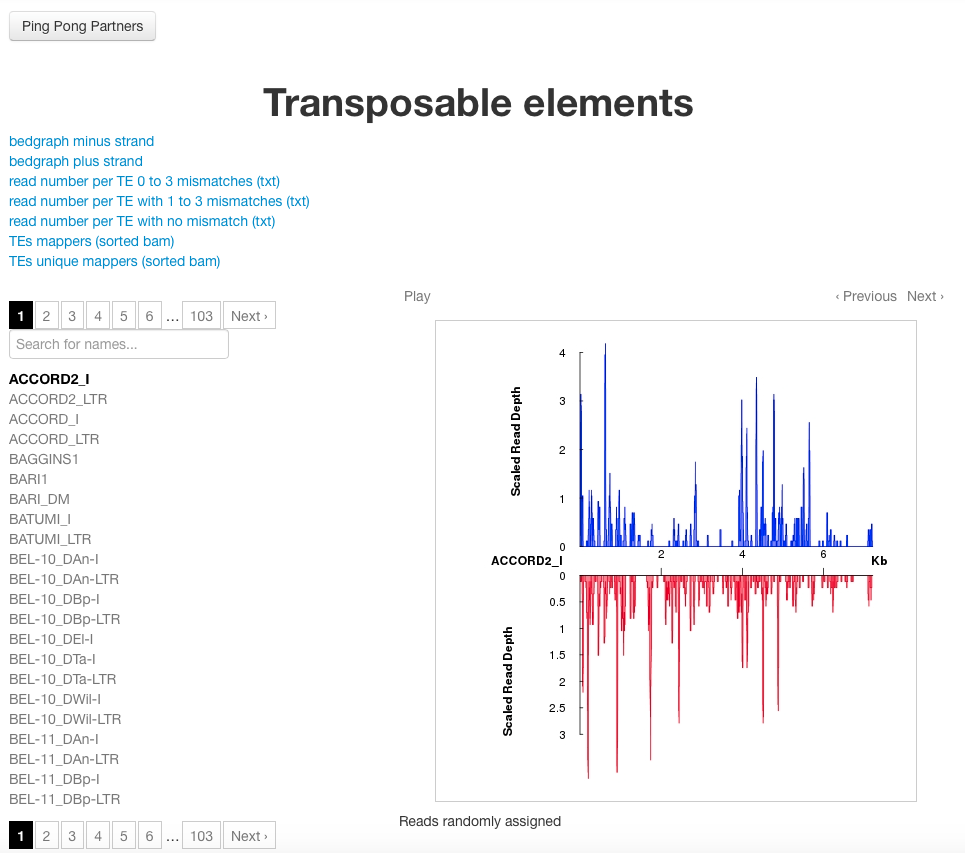


1. The ping-pong signature is analysed for each TE and a sum of all overlaps, the sum of 10-nt-overlaps, the mean, the standard deviation, the z-score and the p-value for each TE are summarized in a table.


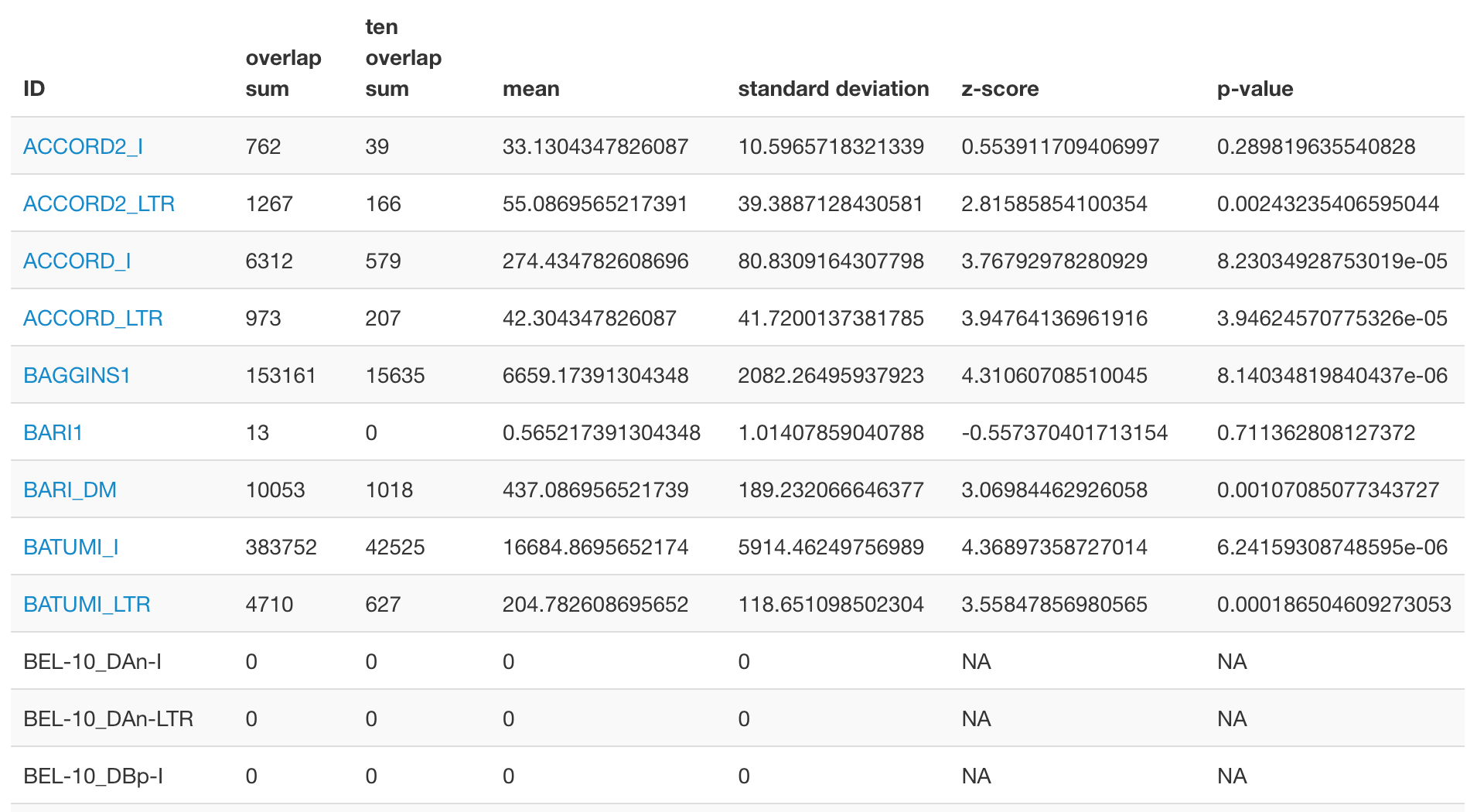


1. By clicking on a particular TE in the table, a histogram of the percentage of 5’-overlaps of reads in opposite orientation is accessed. Reads with or without ping-pong partners, in sense and in antisense orientation, can be downloaded for further analysis.


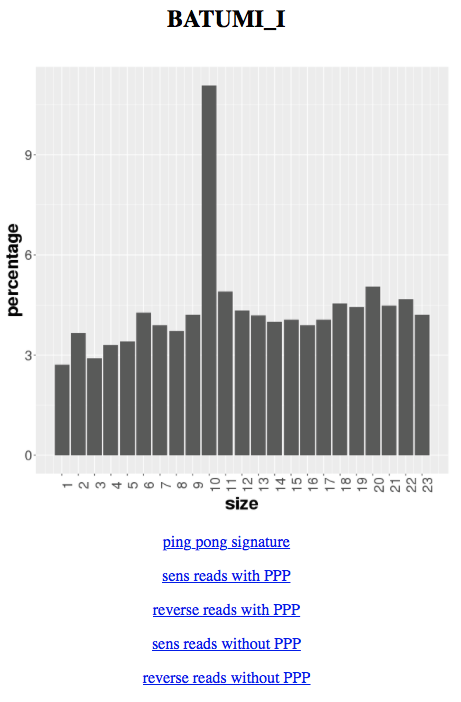


All results can be downloaded, either by a click on the corresponding link in the results folders, or as a whole by clicking on the name of the results item and then clicking the Save icon.

**Results as obtained with the small data files for a quick test:**

1. On the first page:
   - The size distribution of genome-mapped reads and the number of all genome-mappers and of genome-unique mappers (reads that map only once to the genome) can be visualized.


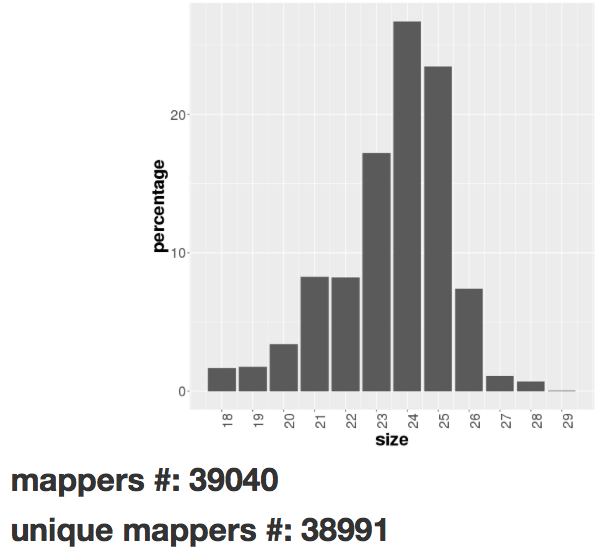


- - Figures show mapping of genome-unique reads ("Uniquely mapped reads") and of all reads randomly assigned ("Reads randomly assigned") to the chromosomes. Small RNAs are mapped on plus and on minus strands, in blue and in red respectively.


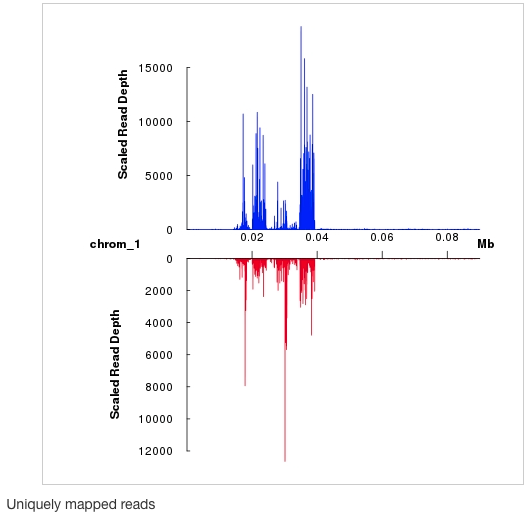

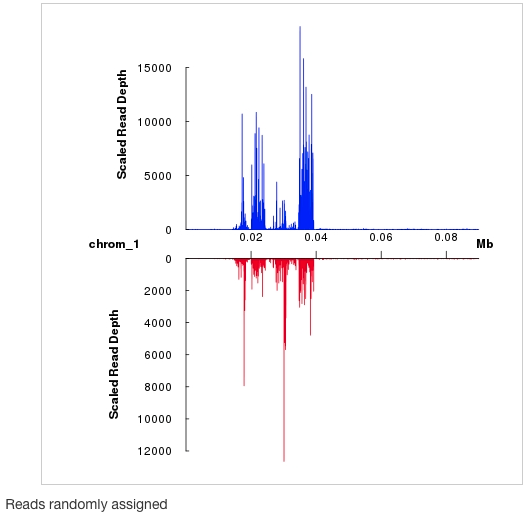


- - The corresponding bam and bedgraph files are accessible *via* the corresponding links on the upper left, as well as the counts of the mappers size distribution and lists of the mapped and unmapped reads.


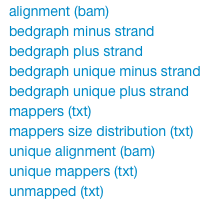


1. The second page is accessible by clicking on "View details" on the first page of results. Then the information for 4 subgroups, Bonafide reads, siRNAs, piRNAs and miRNAs, is accessible. For each subgroup, three distinct analyses are accessible in different folders: for genome-mapping reads, TE-mapping reads and transcript-mapping reads.


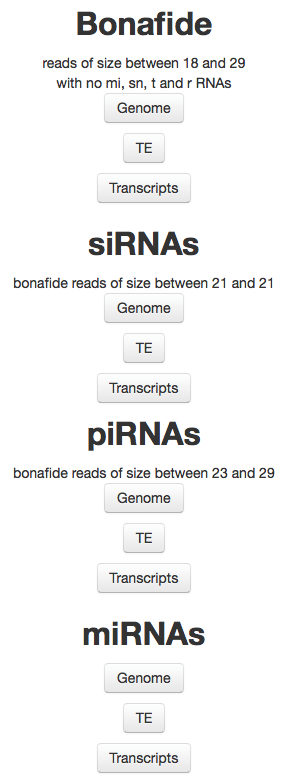


1. By clicking on Genome, TE or Transcripts folders, figures for genome-unique mappers and for all mappers and tables with read counts for the corresponding features (genome, TEs or transcripts) can be visualized, the corresponding sorted BAM files and bedgraphs for the plus and minus strand mappers can be downloaded. To quickly search for a desired TE or contig, a search function is available.


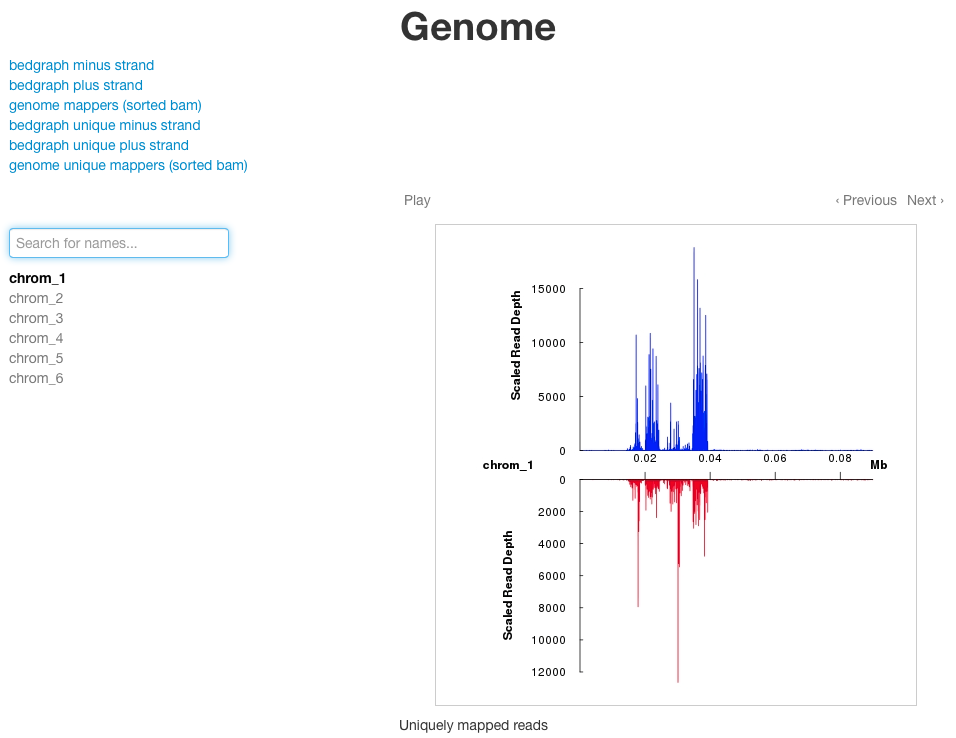

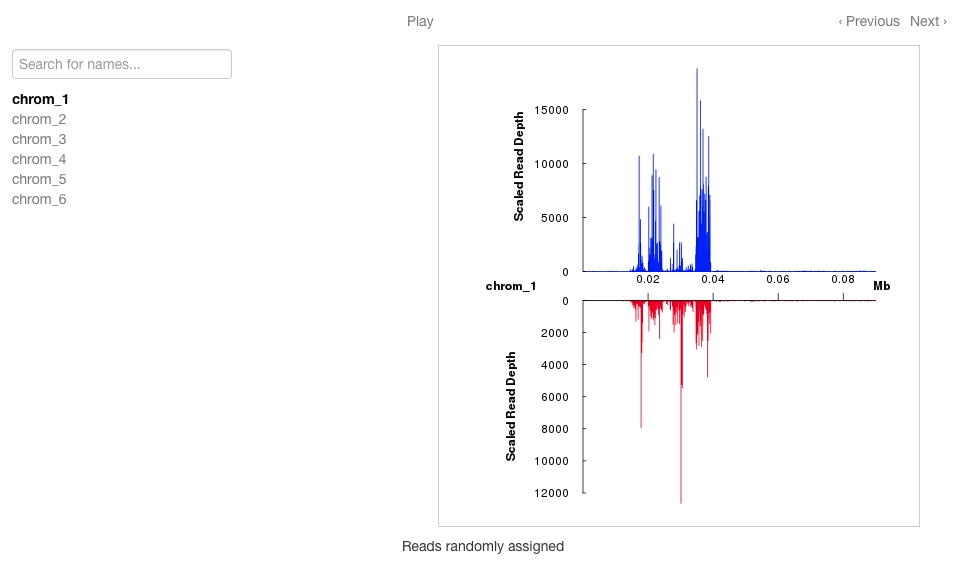


1. The results for Ping-pong signature ("PPPartners") are found only in the "piRNAs" subgroup "TE" folder, by clicking on "Ping Pong Partners".


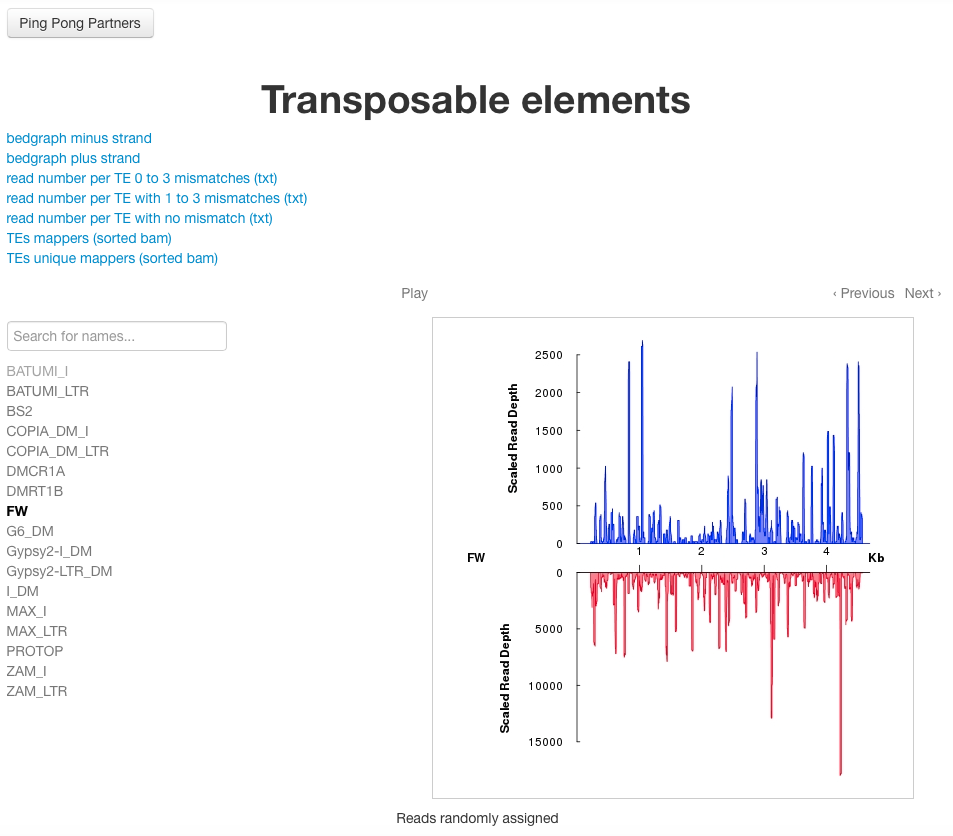


1. The ping-pong signature is analysed for each TE and a sum of all overlaps, the sum of 10-nt-overlaps, the mean, the standard deviation, the z-score and the p-value for each TE are summarized in a table.


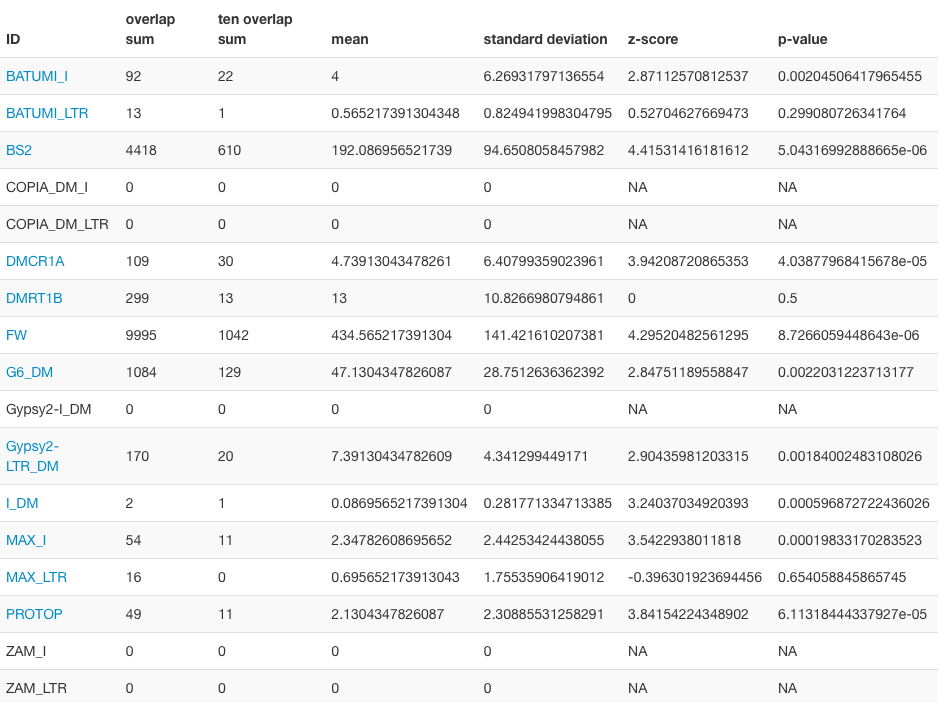


1. By clicking on a particular TE in the table, a histogram of the percentage of 5’-overlaps of reads in opposite orientation is accessed. Reads with or without ping-pong partners, in sense and in antisense orientation, can be downloaded for further analysis.


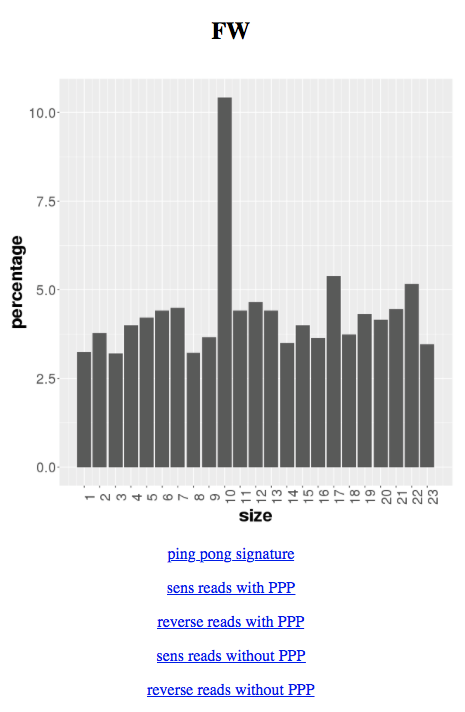


All results can be downloaded, either by a click on the corresponding link in the results folders, or as a whole by clicking on the name of the results item and then clicking the Save icon.
